# How to improve the physical health of people with severe mental illness? A multicentric randomized controlled trial on the efficacy of a lifestyle group intervention

**DOI:** 10.1192/j.eurpsy.2021.2253

**Published:** 2021-11-23

**Authors:** Mario Luciano, Gaia Sampogna, Mario Amore, Ileana Andriola, Pietro Calcagno, Claudia Carmassi, Valeria Del Vecchio, Liliana Dell’Osso, Giorgio Di Lorenzo, Barbara Gelao, Vincenzo Giallonardo, Alessandro Rossi, Rodolfo Rossi, Alberto Siracusano, Andrea Fiorillo, LIFESTYLE Working Group

**Affiliations:** 1 Department of Psychiatry, University of Campania “L. Vanvitelli”, Naples, Italy; 2 Section of Psychiatry, Department of Neuroscience, Ophthalmology, Genetics and Infant-Maternal Science, University of Genoa, Genoa, Italy; 3 Department of Basic Medical Science, Neuroscience and Sense Organs, University of Bari “Aldo Moro”, Bari, Italy; 4 Department of Clinical and Experimental Medicine, University of Pisa, Pisa, Italy; 5 Department of System Medicine, University of Rome Tor Vergata, Rome, Italy; 6 Section of Psychiatry, Department of Biotechnological and Applied Clinical Sciences, University of L’Aquila, L’Aquila, Italy

**Keywords:** Comorbidity, HOMA-IR index, Framingham risk score, RCT, BMI, waist circumference, severe mental disorders, lifestyle

## Abstract

**Background:**

People with severe mental illnesses (SMI) have a mortality rate two times higher compared to the general population, with a decade of years of life lost. In this randomized controlled trial (RCT), we assessed in a sample of people with bipolar disorder, major depressive disorder, and schizophrenia spectrum disorder, the efficacy of an innovative psychosocial group intervention compared to a brief psychoeducational group intervention on patients’ body mass index (BMI), body weight, waist circumference, Framingham and HOMA-IR indexes.

**Methods:**

This is a multicentric RCT with blinded outcome assessments carried out in six Italian university centers. After recruitment patients were randomized to receive a 6-month psychosocial intervention to improve patients’ physical health or a brief psychoeducational intervention. All recruited patients were assessed with standardized assessment instruments at baseline and after 6 months. Anthropometric parameters and blood samples have also been collected.

**Results:**

Four-hundred and two patients with a diagnosis of bipolar disorder (43.3%), schizophrenia or other psychotic disorder (29.9%), or major depression (26.9%) were randomly allocated to the experimental (*N* = 206) or the control group (*N* = 195). After 6 months, patients from the experimental group reported a significant reduction in BMI (odds ratio [OR]: 1.93, 95% confidence intervals [CI]: 1.31–2.84; *p* < 0.001), body weight (OR = 4.78, 95% CI: 0.80–28.27, *p* < 0.05), and waist circumference (OR = 5.43, 95% CI: 1.45–20.30, *p* < 0.05). Participants with impaired cognitive and psychosocial functioning had a worse response to the intervention.

**Conclusions:**

The experimental group intervention was effective in improving the physical health in SMI patients. Further studies are needed to evaluate the feasibility of this intervention in real-world settings.

## Introduction

People with severe mental illnesses (SMI), such as schizophrenia, bipolar disorder, and major depression, are more likely to suffer from obesity, diabetes, dyslipidemias, and metabolic syndrome with an increase of cardiovascular risk [1–7] and a reduced life expectancy of up to 25 years compared with the general population, representing a major public health concern [8–13] and a priority for health agencies and national governments [14].

Several factors can contribute to the poor physical health in this patient population. First, they frequently adopt unhealthy lifestyle behaviors, characterized by lack of physical activity, unhealthy diet (rich in carbohydrate and fat), heavy smoking, and use of alcohol or illicit substances [15–19]. Second, several psychotropic medications have metabolic side effects, further increasing the cardiometabolic risk [20–26]. Third, patients with SMI rarely access screening procedures for physical illnesses due to patients’ lack of motivation and stigma from other physicians [27–30]. Finally, several illness-related factors, including cognitive impairment, reduced psychosocial functioning, social isolation, and self-stigma [31–34], can reduce patients’ autonomy to make decisions about their physical health condition. In fact, SMI patients tend to perceive stigmatizing attitudes from their healthcare providers, and thus are reluctant to seek medical help [5]. Internalized stigma causes social withdrawal which may lead to further reduce check-up visits for physical health [35], and a consequent increase of incidence of cardiovascular diseases [34] and excess mortality [31,36].

The increase in life expectancy of the general population, due to better living conditions and advanced medical care, may increase the prevalence of physical comorbidities, particularly noncommunicable diseases (such as cancer, diabetes, and cardiovascular illness). Despite this, the management of physical comorbidities in SMI patients is still a neglected and understudied area for healthcare professionals, in particular due to the over-fragmentation of medical disciplines [4].

The improvement of patients’ lifestyle behaviors can be associated with a significant improvement in quality of life, with a reduction in morbidity and mortality rate in the long term [37,38]. In the past decades, several supportive interventions, including nutritional and motivational components, have been developed with the aim to modify patients’ lifestyle behaviors [39]. However, evidence to support the efficacy of lifestyle interventions remains scarce [3,39,40]. In fact, while some studies have found that patients receiving a lifestyle intervention report a significant weight loss and a reduction in cardiovascular risk factors [41–43], other studies failed to demonstrate the benefits of such interventions [44]. This discrepancy in findings can partly be attributed to methodological limitations, with many studies carried out on small sample sizes [8], or lacking adequate control groups [10], or with participants who were not overweight or obese.

Other studies have focused on the difficulties in implementing lifestyle interventions in routine clinical care, which are mainly due to the involvement of multidisciplinary teams, the long duration of the intervention, the structured model of the programs, and the high costs of the intervention in the real word practice [41]. Despite this, psychosocial interventions for promoting healthy lifestyle behaviors among people with SMI are effective and very well received by patients [45,46].

We carried out a multicentric study, coordinated by the Department of Psychiatry of the University of Campania “L. Vanvitelli” and carried out in six university sites, with the aim to assess the efficacy of a new psychosocial group intervention (the LIFESTYLE intervention) in a sample of patients with SMI in the real world. The main innovations of our intervention include: (a) the use of elements derived from classical psychoeducation, motivational interview, and cognitive-behavioral therapy; (b) the adoption of a comprehensive approach focusing on all aspects of unhealthy lifestyles, and not addressing only unhealthy diet; and (c) the provision of the intervention to mixed diagnostic groups of patients.

The primary aim of the study is to evaluate the efficacy of the LIFESTYLE intervention in terms of reduction of body mass index (BMI) at 6 months of follow-up, compared to a brief psychoeducational group intervention (control intervention). The secondary aims include the reduction of the Framingham and HOMA-IR (homeostasis model assessment of insulin resistance) indexes, waist circumference, and of comorbidity and severity indexes at the cumulative illness rating scale (CIRS). Moreover, we aimed to explore the impact of several illness-related variables, such as cognition and psychosocial functioning, on the efficacy of the intervention. We hypothesize that at 6 months patients receiving the experimental intervention will observe a reduction of at least one point of BMI and that patients with a worse psychosocial and cognitive functioning will benefit less from the experimental intervention.

## Methods

The LIFESTYLE trial is a multicentric, randomized controlled trial (RCT) with blinded outcome assessments, carried out in the outpatient units of the University of Campania “Luigi Vanvitelli” in Naples, University of Bari, University of Genova, University of L’Aquila, University of Pisa, and University of Rome-Tor Vergata, and funded by the Italian Ministry of Education, Universities and Research within the framework of the “Progetti di Rilevante Interesse Nazionale (PRIN).” Each center was expected to recruit 70 patients, 35 per each arm, with a total sample size of 420 participants.

Eligible patients were identified by their clinicians and referred to the LIFESTYLE research staff by phone, email, or in-person meeting. After the informed consent, all patients were randomly allocated to the two arms by the coordinating center. The randomization procedure was stratified according to center, age, gender, and educational level with a 1:1 ratio.

To be included in the study patients had to fulfill the following inclusion criteria: (a) age between 18 and 65 years; (b) diagnosis of schizophrenia, schizoaffective disorder, delusional disorder, other psychotic disorders, major depressive disorder, or bipolar disorder according to the Diagnostic and Statistical Manual of Mental Disorders, Fifth Edition (DSM-5) and confirmed by the Structured Clinical Interview for DSM-5 (SCID-5) [47]; (c) ability to provide written informed consent; (d) BMI ≥ 25; (e) in charge to the local mental health unit for at least 3 months before the inclusion in the study. Patients were excluded in case of: (a) inability to perform moderate physical activity (i.e., walking at least 150 min per week, or 75 min of vigorous activity twice a week, according to the guidelines of the Italian Ministry of Health); (b) pregnancy or breast-feeding; (c) intellectual disability or severe cognitive impairment; and (d) worsening of clinical status or hospital admission in the previous 3 months. All recruited patients provided written informed consent to participate in the study.

Researchers and statisticians involved in assessments of patients were blinded to patient’s allocation. This study was conducted in accordance with globally accepted standards of good practice, in agreement with the Declaration of Helsinki and with local regulations. The study protocol was formally approved by the Ethics Committee of the Coordinating Center on January 24, 2017 (approval number: prot. 64).

### Interventions

#### Arm I: Lifestyle psychosocial group intervention

The theoretical background of the experimental intervention includes techniques derived from classic psychoeducation [48,49], motivational interview [50–52], and cognitive-behavioral therapy [53]. The intervention was developed following the guidelines on the management of physical health in people with mental disorders produced by the World Health Organization [54,55], the European Association for the Study of Diabetes [56], the European Society of Cardiology [57], and the European Psychiatric Association [58].

The full methodology adopted to develop the intervention has been reported in detail elsewhere [59]. The intervention lasted 6 months and was administered to groups of 5–10 patients every 7–10 days. The following topics were covered during the sessions: (a) healthy diet; (b) physical activity; (c) smoking habits; (d) medication adherence; (e) risky behaviors; and (f) promotion of circadian rhythms. Each module included the following components: (a) education on the risks and benefits of each lifestyle behavior; (b) provision of practical strategies to reduce unhealthy behaviors; and (c) for each participant, identification of personal life goals, motivation to change, and problem-solving strategies. During sessions, working groups and active interaction among participants were developed to stimulate discussion. At the end of each meeting, a 20-min session of moderate physical activity was scheduled.

#### Arm II: Brief psychoeducational group intervention

The brief psychoeducational group intervention consisted of five weekly sessions administered to groups of 5–10 patients on: (a) healthy lifestyle; (b) early detection of clinical relapses; (c) effects of pharmacological treatment and management of side effects; (d) stress management techniques; and (e) problem-solving techniques. Manuals were developed for both interventions in order to ensure treatment fidelity among the centers.

#### Training of mental health professionals

Three mental health professionals (at least one being a psychiatrist) per each center participated to a 5-day training course on the two interventions. All mental health professionals received regular phone and e-mail supervisions during the whole study period. Moreover, a site visit was organized by the coordinating center to guarantee an in vivo supervision.

#### Assessment times and instruments

A further 2-day meeting was organized for the use of assessment instruments, according to study protocol.

Researchers participating to the study were different from those running the interventions and were blinded to patient allocation.

Patients were assessed at baseline (T0) and 6 months after randomization (T1). Patients’ diagnosis was confirmed through the SCID-5.

Patients’ psychiatric symptoms and psychosocial functioning were assessed by: (a) the Brief Psychiatric Rating Scale (BPRS) [60], a semi-structured 24-item interview on psychopathological status. Items are grouped in four subscales: positive symptoms, negative symptoms, depressive-anxiety symptoms, and manic/hostility symptoms; (b) the Personal and Social Performance Scale (PSP) [61], a 100-point single-item rating scale, subdivided into 10 equal intervals; and (c) the Measurement and Treatment Research to Improve Cognition in Schizophrenia (MATRICS) Consensus Cognitive Battery (MCCB)—brief version, which includes the MATRICS Consensus Trail Making Test—Part A, Brief Assessment of Cognition in Schizophrenia: Symbol Coding, Category Fluency-Animal Naming [62,63].

Patients’ physical health was assessed with the following instruments: (a) the CIRS [64], a 14-item questionnaire exploring presence and severity of physical comorbidities; (b) an anthropometric schedule with information on weight, height, BMI, waist circumference, blood pressure, resting heart rate, high-density lipoprotein, low-density lipoprotein, overall cholesterol levels, blood glucose, triglycerides, and blood insulin; (c) the homeostasis model assessment of insulin resistance (HOMA-IR), calculated as follows: fasting insulin (mg/dL) × fasting glucose (mmol/L)/405 [65]; (d) the Framingham risk score (FRS) for the evaluation of cardiovascular risk [66].

The inter-rater reliability of researchers was tested through the Cohen’s Kappa coefficient, which was satisfactory for both the PSP (*K* value = 0.918) and the BPRS (*K* value ranging from 0.835 to 0.972). A 100% agreement rate was found for the SCID-5 diagnoses.

### Statistical analyses

Statistical analyses were conducted according to the “Intention To Treat” principle. Missing data were handled using the Last Observation Carried Forward. Descriptive statistics (frequency table, means and standard deviation) were calculated for both experimental and control groups at baseline and at the end of the intervention. Differences in sociodemographic and clinical characteristics among the two groups at baseline and at the end of the intervention were tested using *χ* ^2^ or *t*-test for independent samples, as appropriate. The impact of the interventions on physical health related domains was explored by the Student’s *t*-test for paired sample in each group.

Generalized estimating equation (GEE) models were used for evaluating the impact of the experimental intervention on the primary outcome (i.e., reduction of BMI at the end of the intervention). GEE models allow estimation of population-averaged models in repeated-measures data. Control vs. intervention interaction terms assessed changes between groups over time; Wald tests determined whether joint effects of time-by-group equaled zero. Age and center were included as time-invariant covariates; time-varying covariates included medications, cognitive functioning, age, gender, and type of mental disorder diagnosis. We used GEE models with a normal distribution and identity link. We report covariate-adjusted results using robust estimates of standard errors. All models were adjusted for diagnosis, pharmacological treatments, duration of illness, and educational level. Pharmacological treatments and psychiatric diagnoses have been included in the regression models as dummy variables (i.e., mood stabilizers, tricyclic antidepressants, new-generation antidepressants, first- and second-generation antipsychotics, depressive disorder, bipolar disorders, psychosis).

The Statistical Package for Social Sciences (SPSS), version 17.0 (SPSS Inc., Chicago), and STATA, version 15 (StataCorp LLC, College Station, TX), were used for statistical analyses. For all analyses, the level of statistical significance was set at *p* < 0.05.

## Results

A total of 401 patients agreed to participate in the study and were randomly allocated to receive the experimental or the control intervention (206 from the experimental group and 195 from the control group). Two hundred and twenty-four patients (112 in the experimental and 112 in the control group) did not complete the intervention due to: practical difficulties in reaching the study site (27%), not anymore in charge to the local mental health center (25%), exacerbation of psychiatric symptoms (20%), and lack of interest (18%). Therefore, the final sample consisted of 177 patients (94 in the experimental and 83 in the control group).

### Sociodemographic and clinical characteristics of the two samples

Of the 402 recruited patients, 43.3% had a diagnosis of bipolar disorder, 29.9% of psychosis, and 26.9% of major depression. Patients were mainly female (57%), with an average age of 45.6 ± 11.8 years and educational level of 11.7 ± 2.9 years. Of them 28.6% were married (Table 1). All patients were treated with at least one psychotropic drug: 35% of the sample was treated with one pharmacological agent, 39% with two, 21% was taking three different drugs and up to 5% was treated with four different psychotropic drugs.

**Table 1.** Sociodemographic and clinical characteristics of the sample (*N* = 402).

Gender, female, % (N)	57 (227)
Age, *M* (SD)	45.8 (11.8)
Living situation, % (*N*)	
Alone	71.4 (287)
Married	28.6 (115)
Years of education, *M* (SD)	11.69 (2.9)
Employed, yes % (*N*)	35.7 (143)
Diagnosis, % (*N*)	
Bipolar I disorder	31.3 (125)
Bipolar II disorder	11.8 (47)
Schizophrenia	16.8 (67)
Schizoaffective disorder	13.0 (12.9)
Major depression	27.1 (108)
Years in charge to the mental health service, *M* (SD)	5.9 (6.9)
Duration of illness, *M* (SD)	15.6 (11.3)
Number of hospitalization, *M* (SD)	2.8 (5.1)
Suicide attempts, *M* (SD)	1.8 (1.6)
BPRS, positive symptoms, *M* (SD)	5.4 (2.1)
BPRS, negative symptoms, *M* (SD)	7.7 (3.1)
BPRS, depressive/anxiety symptoms, *M* (SD)	8.8 (3.1)
BPRS, manic/hostility symptoms, *M* (SD)	4.7 (1.9)
B-MCCB, symbol coding, *M* (SD)	36.9 (50.3)
B-MCCB, animal naming, *M* (SD)	20.3 (49.3)
B-MCCB trial making test A, *M* (SD)	69.1 (127.9)
PSPS, total score, *M* (SD)	65.5 (15.1)
Typical antipsychotics, yes % (*N*)	22.5 (90)
Atypical antipsychotics, yes % (*N*)	59 (236)
Antidepressants, yes % (*N*)	51.5 (205)
Benzodiazepine, yes % (*N*)	47.1 (189)
Mood stabilizers, yes % (*N*)	65.8 (264)

Abbreviations: B-MCCB: Brief MATRICS Consensus Cognitive Battery; BPRS: Brief Psychiatric Rating Scale; PSPS: Personal and Social Performance Scale; SD, standard deviation.

Patients had a mean body weight of 91.4 kg (±17.4), with a BMI of 32.5 (±5.5) and a waist circumference of 109.3 cm (±14.2). The systolic and diastolic blood pressure were 125.6 (±13.53) and 80.7 (±9.0), respectively. The mean score at the CIRS severity index was 0.5 (±4.6) and the mean score at the CIRS comorbidity index was 0.3 (±1.4). The mean FRS was 9.8 (±4.5) and the mean HOMA-IR index was 4.9 (±11.6) (Table 2).

**Table 2.** Comparisons of health-related domains in the two groups.

	Experimental treatment (*N* = 94)		Control group (*N* = 83)	
	Baseline	T1	Baseline	T1
BMI, kg/m^2^, *M* (DS)	32.17 (5.25)	30.60 (4.50)**	32.88 (5.78)	32.84 (6.19)
Body weight, *M* (DS)	91.78 (17.2)	87.59 (15.34)*	92.13 (17.6)	92.27 (18.28)
Waist circumference, *M* (DS)	108.64 (14.37)	105.89 (12.8)**	109.88 (13.66)	109.51 (14.0)
CIRS, severity index, *M* (SD)	0.30 (0.28)	0.29 (0.23)	0.30 (0.35)	0.28 (0.28)
CIRS, Comorbidity index, *M* (SD)	0.33 (1.57)	0.30 (1.02)	0.29 (1.21)	0.30 (0.29)
Waist circumference, cm, *M* (SD)	108.65 (14.37)	107.89 (12.80)	109.89 (13.65)	109.52 (13.97)
Systolic blood pressure, *M* (SD)	125.58 (13.64)	124.24 (11.80)	125.64 (13.48)	124.93 (11.94)
Diastolic blood pressure, *M* (SD)	81.15 (9.33)	79.96 (7.47)	80.34 (8.61)	78.84 (11.59)
Blood glucose, mg/dL, *M* (SD)	95.33 (20.90)	96.04 (25.94)	95.56 (32.31)	99.13 (42.22)
Total cholesterolemia, mg/dL, *M* (SD)	192.71 (42.05)	188.29 (36.00)	186.87 (39.62)	187.40 (41.91)
Serum triglycerides, mg/dL, *M* (SD)	180.86 (156.16)	172.74 (129.92)	161.04 (87.73)	154.44 (81.18)
Framingham risk score, total score, *M* (SD)	9.78 (8.10)	8.91 (5.94)	8.88 (6.77)	8.10 (5.64)
HOMA-IR index, *M* (SD)	4.31 (5.65)	4.14 (4.90)	4.66 (5.92)	6.42 (11.08)

Abbreviations: BMI, body mass index; CIRS, Cumulative Illness Rating Scale; DS, Standard Deviation; HOMA-IR, homeostatic model assessment of insulin resistance; SD, standard deviation.

* *p* < 0.05.

** *p* < 0.001.

No statistically significant differences were found in sociodemographic and clinical characteristics as well as in anthropometric and metabolic parameters between experimental and control groups.

### Efficacy of the experimental intervention

At the univariate analyses, patients from the experimental group reported a significant reduction in BMI and body weight (32.2 ± 5.2 at T0 vs. 30.6 ± 4.5 at T1 and 91.8 ± 17.2 vs. 87.6 ± 15.3, *p* < 0.01, respectively). Moreover, at the end of the intervention, patients from the experimental group reported a mean reduction in waist circumference of 2.75 cm (*p* < 0.05) (Table 2).

The GEE model showed a significant effect of the experimental intervention on BMI. In particular, at the end of the intervention, patients receiving the experimental intervention had a BMI reduction of almost two points (odds ratio [OR]: 1.93, 95% confidence intervals [CI]: 1.31–2.84; *p* < 0.001). Other factors positively impacting the reduction of BMI were female gender (OR: 0.32, 95% CI: 0.12–0.84), a better cognitive functioning (B-MCCB symbol coding, OR: 0.99, 95% CI: 0.94–1.03, *p* < 0.001; B-MCCB animal naming, OR: 0.94, 95% CI: 0.85–1.03, *p* < 0.001; B-MCCB trial making test A, OR: 0.99, 95% CI: 0.90–1.02, *p* < 0.001), and a better psychosocial functioning (PSP total score: OR: 0.73, 95% CI: 0.98–1.02, *p* < 0.05) (Table 3).

**Table 3.** Generalized estimating equation (GEE) models.

	BMI	Weight	CIRS severity index	CIRS comorbidity index	FRS total point	HOMA-IR index	Waist circumference
	OR (95% CI)	OR (95% CI)	OR (95% CI)	OR (95% CI)	OR (95% CI)	OR (95% CI)	OR (95% CI)
Experimental treatment	1.93 (1.31–2.84)***	4.78 (0.80–28.27)*	0.98 (0.94–1.01)	0.98 (0.90–1.07)	0.60 (0.30–1.12)	1.26 (0.48–3.26)	5.43 (1.45–20.30)*
Gender, female	0.32 (0.12–0.84)*	5.02 (5.32–6.12)***	0.98 (0.94–1.02)	0.95 (0.87–1.03)	7.28 (3.78–14.03)***	2.13 (0.73–6.21)	5.80 (3.94–6.01)*
Age	1.00 (0.95–1.05)	0.95 (0.78–1.10)	1.01 (1.00–1.01)***	1.0 (0.9–1.0)*	1.43 (1.38–1.48)***	1.01 (0.99–1.05)	1.22 (1.13–1.32)***
PSP, total score	0.73 (0.98–1.02)*	0.88 (0.75–1.4)***	0.99 (0.99–1.0)*	0.99 (0.99–1.0)**	0.98 (0.96–1.00)	0.94 (0.91–0.98)**	0.99 (0.98–0.99)**
B-MCCB symbol coding	0.98 (0.94–1.03)***	0.95 (0.80–1.13)**	1.00 (1.00–1.01)	0.99 (0.99–1.00)	0.97 (0.94–1.00)	0.98 (0.92–1.06)	1.00 (0.99–1.01)
B-MCCB category fluency: animal naming	0.94 (0.85–1.03)***	1.03 (0.74–1.4)	0.99 (0.98–1.00)	0.99 (0.98–1.00)	0.99 (0.97–1.00)	0.99 (0.97–1.01)	0.98 (0.97–0.98)***
B-MCCB trial making test A	0.99 (0.90–1.02)***	0.96 (0.76–1.03)**	0.99 (0.99–1.00)	0.99 (0.99–1.00)	0.99 (0.97–1.00)	0.99 (0.97–1.01)	1.00 (0.98–1.0)

GEE have been adjusted for diagnosis, pharmacological treatments, duration of illness, and years of education.

Abbreviations: B-MCCB, Brief MATRICS Consensus Cognitive Battery; BMI, body mass index; CI, confidence intervals; CIRS, Cumulative Illness Rating Scale; FRS, Framingham risk score; HOMA-IR, homeostatic model assessment of insulin resistance; PSP, Personal and Social Performance.

* *p* < 0.01.

** *p* < 0.01.

*** *p* < 0.001.

The GEE model also showed a significant effect of the experimental intervention on body weight and waist circumference. In fact, people receiving the experimental intervention had a 4.78 times increase in the probability to significantly reduce their body weight (OR = 4.78, 95% CI: 0.80–28.27, *p* < 0.05) and a 5.43 times increase in the probability to significantly reduce waist circumference (OR = 5.43, 95% CI: 1.45–20.30, *p* < 0.05). Moreover, several factors including male gender (OR = 5.02, 95% CI: 5.32–6.12, *p* < 0.001), high cognitive functioning (B-MCCB symbol coding, OR: 0.95, 95% CI: 0.80–1.13, *p* < 0.01; B-MCCB Trial making test A, OR: 0.96, 95% CI: 0.76–1.03, *p* < 0.01), and better psychosocial functioning (PSP total score, OR: 0.88; 95% CI: 0.75–1.4) had a positive impact on the reduction of body weight. Similarly, male gender (OR = 5.80, 95% CI: 3.94–6.01, *p* < 0.05), age (OR: 1.22, 95% CI: 1.13–1.32, *p* < 0.001), a better psychosocial functioning (PSP total score, OR: 0.99, 95% CI: 0.98–0.99, *p* < 0.01), and a better cognitive functioning (B-MCCB animal naming, OR: 0.98, 95% CI: 0.97–0.98, *p* < 0.001) had a significant impact on the efficacy of the intervention.

At the GEE models, the experimental intervention did not have a significant impact on the probability of reducing the FRS, the HOMA-IR index, and the CIRS severity indexes (Table 3). In all GEE models, the effect of the intervention was controlled for several confounding variables, including diagnosis, pharmacological treatments, duration of illness, and years of education.

## Discussion

The LIFESTYLE trial represents the first multicenter study carried out in Italy aiming to assess the impact of a psychosocial intervention targeting lifestyle behaviors in people with SMI in real-world settings. Effective interventions for improving physical health in people with severe mental disorders are highly needed in order to reduce the premature mortality in this vulnerable group of people [11].

The main aim of the study was to evaluate the efficacy of the LIFESTYLE intervention, compared to a brief educational program, in reducing BMI of overweight patients with SMI. Our findings confirmed our hypothesis, with a significant reduction in BMI, body weight, and waist circumference in patients receiving the experimental intervention. Our results also support the recent finding that people with SMI can achieve healthy lifestyle through the provision of behavioral programs [67].

We selected the BMI as primary outcome since it is a reliable and easy to assess anthropometric parameter that can provide stable and useful information compared to body weight changes [59]. In the present trial, treated patients reported a significant reduction in BMI of almost two points, confirming the efficacy of the intervention. When comparing our results with those derived from other non-pharmacological interventions for lifestyle behaviors, we found higher weight losses in the ACHIEVE [42] and in the STRIDE [68] trials, of 3.2 and 4.4 kg, respectively. On the contrary, other RCTs, such as the STEPWISE [69] and CHANGE trials [44,70], did not find any effect of the intervention on weight losses [71]. For this reason, we believe that a reduction of BMI may be a more reliable outcome measure, which should be adopted to evaluate the efficacy of lifestyle interventions [16].

Another significant finding of our study is the efficacy of the experimental intervention on waist circumference. Differently from BMI and body weight, waist circumference is a specific proxy measure of abdominal obesity, which is highly correlated with the lifetime risk of cardiovascular disorders [72].

We had a quite high drop-out rate both in the experimental and in the control group. However, this finding is in line with previous trials on behavioral interventions, where attrition rates of up to 40% were reported, even with brief interventions [44,73]. Moreover, this finding is also similar to that reported with other psychosocial interventions for people with SMI [44,70,73]. Several strategies could improve the rate of participants who complete the psychoeducational intervention; in particular, such electronic reminders (e.g., phone calls, emails, instant messages), availability of dedicated staff members and of rooms/spaces to run the intervention, and more time for professionals to run the intervention [49,74,75]. Future implementation strategies should include web-based components with the integration of smartphone apps and wearable devices, for increasing real-time interactions with participants [76].

The efficacy of the experimental intervention was influenced by several clinical domains, such as poor cognitive performance and impaired psychosocial functioning, but not by others, such as the levels of psychiatric symptoms and the number of hospitalizations. It is likely that patients with SMI presenting with a worse performance on recall, verbal, and working memory, have a high BMI and a significantly high risk for cardiovascular diseases [37, 77–79] due to cognitive deficits in recalling the medical appointments and in taking medications properly. However, only a few studies have assessed the impact of cognitive performance on the efficacy of behavioral intervention focused on weight reduction, and this should be further explored in subsequent studies. Moreover, patients with cognitive deficits may be less motivated to participate and to be actively involved in interventions for the promotion of their physical health [80,81]. Therefore, cognitive deficits may represent the mechanisms underlying poor physical health in patients with SMI, independently from the main psychiatric diagnosis and symptoms.

The relationship between physical health and patients’ social functioning has been explored only in a few studies. Patients with reduced social functioning have a higher risk of developing physical illnesses due to poor skills in help seeking [82] and to low levels of physical and daily activities [83]. Patients with poor psychosocial functioning have a reduced autonomy in performing daily tasks, in engaging in complex behavioral changes, and in participating in social activities, highlighting that an improvement of psychosocial functioning may result in an improvement of physical health.

In this article, we have not evaluated the differences between the three diagnostic groups in response to the psychosocial intervention; however, the multivariable models have been adjusted according to patients’ diagnoses, and therefore we can assume that there is not a diagnosis effect, but this finding needs confirmation [37], supporting the hypothesis of a transdiagnostic approach to mental healthcare [84–86].

The main strengths of the LIFESTYLE intervention, compared to already available interventions for people with SMI, include the following: (a) the integration of psychoeducational, motivational, and cognitive-behavioral techniques, with the motivational component resulting as one of the most important strategies to support behavioral changes and promote weight loss [87–89]; (b) a comprehensive approach addressing almost all components of healthy living, such as healthy diet, reduction of sedentary behaviors, promotion of physical exercise, retaining from tobacco smoking and risky behaviors, improving sleep hygiene and promoting the regularization of circadian rhythms, and increasing medication adherence; (c) the provision of the intervention in a group format for patients with different diagnoses, which allowed us to compare patients’ experiences and lifestyles not directly linked with the mental disorder; and (d) the inclusion of the recommendations on the management of physical health provided by the World Health Organization, the European Association for Study of Diabetes, the European Society of Cardiology, and the European Psychiatric Association.

Among the study limitations, we must acknowledge the high drop-out rate, which reduces the magnitude of our results. However, the final sample size can be considered satisfactory if compared with already available studies, also because there were no differences at baseline between completers and drop-outs. Furthermore, we did not assess the time of exposure to psychotropic agents, but only the class of pharmacological agents prescribed during the study. However, the multivariable models were controlled for the impact of pharmacological treatments and only stable patients already in charge to the local mental health center for at least 3 months have been included in the study in order to overcome the possible bias due to the effect of medications. Finally, the efficacy of the intervention has been tested only at 6-month follow-up; however, according to the study protocol [59] we aim to assess the long-term efficacy of the intervention at 12 and 24 months of follow-up. Another possible limitation is the adoption of the BPRS to assess the severity of clinical symptoms. We are aware that this tool may have not captured some disease-specific clinical features. However, the BPRS is a well-known instrument frequently used in ordinary psychiatric settings, and it can be used by mental health professionals with different background and following a relatively short training. Another possible limitation is the recruitment of patients with a BMI≥25, which may have reduced the generalizability of our findings. We recruited overweight or obese patients in order to select those at higher risk of developing comorbidities or with compromised lifestyle behaviors already present at the beginning of the intervention. However, next steps would be to provide the intervention regardless the BMI, in order to test its efficacy as a preventive intervention. Finally, it must be acknowledged that all patients were receiving a pharmacological treatment, which can have metabolic side-effect profiles, leading to weight gain, increase in BMI and waist circumference, and alterations in lipid and glucose profiles. However, in order to overcome the possible bias due to the effect of medications, only patients in a stable phase and on a stable medication regimen for at least 3 months have been included in the study. Moreover, we have also controlled the multivariable models for the effect of pharmacological treatments. In our real-world sample 26% of patients received three or more psychotropic drugs, which can be defined as “polypragmasy,” that is the desire to enhance the efficiency of treatment and to help the patient to recover leading to the use of a large number of medications. Such data should be carefully considered in order to improve the good clinical practice in everyday real-world mental health services. Clinicians should be aware that the prescription of polypharmacotherapy can have a negative impact on the patient’s physical health, with a reduction of tolerability of each specific prescribed medication and with the risk of further reduce the efficacy of psychosocial interventions.

Our findings confirm that nonpharmacological interventions improve the physical health of overweight patients with SMI in routine clinical care. In fact, in order to improve the implementation of our approach on a large scale, we decided not to include dieticians, physical trainers, or other professionals not directly present in mental health centers. Moreover, the approach can be provided to the majority of patients with SMI, since its efficacy has been independent from main diagnosis of patients.

These interventions should be better tailored on the unmet needs of patients [90], as well as on the improvement of cognitive deficits and social functioning. New strategies to provide the interventions [91], including the use of new technologies and online sessions [92–94], could further improve the acceptability and the feasibility of the intervention in real-world settings.

## Data Availability

The data that support the findings of this study are available from the corresponding author upon request.
